# Landscape agricultural simplification correlates positively with the spatial distribution of a specialist yet negatively with a generalist pest

**DOI:** 10.1038/s41598-019-57077-4

**Published:** 2020-01-15

**Authors:** Zhaoke Dong, Qingqing Zhang, Lili Li, Zengbin Lu, Chao Li, Fang Ouyang, Teja Tscharntke, Yi Yu, Xingyuan Men

**Affiliations:** 1 Institute of Plant Protection, Shandong Academy of Agricultural Sciences, Ji’nan, 250100 China; 2 Key Laboratory of Integrated Crop Pest Management of Shandong Province, College of Plant Health and Medicine, Qingdao Agricultural University, Qingdao, 266109 China; 3 Maize Research Institute, Shandong Academy of Agricultural Sciences, Ji’nan, 250100 China; 4 State Key Laboratory of Integrated Management of Pest Insects and Rodents, Institute of Zoology, Chinese Academy of Sciences, Beijing, 100101 China; 5 Agroecology, Department of Crop Science, Georg-August-University, Grisebachstrasse 6, 37077 Göttingen, Germany

**Keywords:** Agroecology, Entomology

## Abstract

Agricultural expansion at the cost of natural or semi-natural habitats is simplifying human-dominated landscapes. As croplands provide a large resource of food to herbivores, pest damage may increase, but such large-scale patterns across regions are little known. Here, we used two years of maize field data from 102 counties (each 1318 km^2^ on average) across Shandong Province in China to study the spatial distribution of two major co-occurring maize pests: the putative habitat specialist the Asian Corn Borer (*Ostrinia furnacalis*) (ACB) and the generalist Yellow Peach Moth (*Conogethes punctiferalis*) (YPM). We used Spatial Analysis by Distance Indices (SADIE) to assess the spatial distribution patterns of these pests and their relation to landscape factors. In both 2016 and 2017, the aggregation and abundance of the ACB was positively correlated with the proportion of maize on the county level, whereas the YPM exhibited the opposite pattern, i.e., a negative correlation with maize proportion. The ACB abundance was below the economic threshold level when maize was <31% in 2017, whereas the YPM abundance was below the threshold when maize was >27% (in 2016) or 23% (in 2017). Maize plant presence was the main determinant of the abundance of the ACB, while the YPM appeared to benefit from further resources in non-crop habitats. These contrasting distribution patterns suggest that the two pests are driven by their different resource requirements. In more diversified landscapes, pest control may need to focus primarily on the generalist consumer, the YPM, whereas in maize-dominated landscapes, the specialist consumer, the ACB, is dominant and needs attention.

## Introduction

Agricultural intensification is one of the main drivers of landscape changes. Over the past few decades, agricultural expansion has transformed large areas of natural or semi-natural habitats into cultivated lands^1,2^. The conversion of naturally occurring plant communities into monocultures increases the risk of biodiversity loss and pest outbreaks^3,4^. The simplification of the agricultural landscape is widely expected to exacerbate yield losses to pest species^5–8^.

Previous studies of the effect of landscape simplification have largely focused on the impacts on natural enemies^9,10^. It is often assumed, but rarely empirically shown, that the difference in pest density across landscapes is the result of a change in the impact of natural enemies (top-down control). However, pest herbivores can respond directly to the landscape changes associated with landscape simplification via bottom-up control^11^. Theoretical studies suggest that increases in the size, density, and connectivity of host crop patches facilitate the movement and establishment of crop pests, leading to higher pest pressure^12^. In contrast, empirical studies show that the effects of increasing cultivated or host crop areas on pest abundance are either positive or negative, with no clear trend (reviewed by Veres *et al.*^13^). These contrasting results may be related to the differences between ecological specialists and generalists, which may respond to host patch configuration differently.

The specialist-generalist concept has been used in predicting the response of species to landscape changes^14^. Ecological specialists are expected to benefit from homogeneous environments, while ecological generalists should benefit from heterogeneous environments^14^. Jonsen and Fahrig^15^ found that generalist herbivores respond positively to landscape diversity, while specialists do not. A specialist may be positively related to the amount of cultivated area of its food resource at the landscape scale (e.g., Boiteau *et al*.^16^). Specialist herbivores are more susceptible to habitat loss than generalists^17^. Steffan-Dewenter and Tscharntke^18^ found that the abundance of specialist butterfly species tended to decline with decreasing calcareous grassland fragment size, whereas the abundance of generalists showed the opposite pattern due to the accumulation of individuals from the surrounding landscape matrix. Östergård and Ehrlén^19^ noted that a specialist pre-dispersal seed predator associated with *Lathyrus vernus* (Fabaceae) was more strongly influenced by host plant population size than a generalist seed predator. Specialist and generalist consumers should thus exhibit very different responses to landscape changes.

Knowledge of the spatial distribution of pests in agroecosystems is critical to their effective management. However, while most studies on spatial distribution have only been carried out at the farm scale, studies conducted at the landscape scale or even more, the regional scale across a large area are critical for our understanding of the large-scale effects of habitat change on pest populations. The structure of the landscape may influence the movement behavior of insects, which, in turn, can influence demography at the landscape level. There is a marked absence of empirical work examining the effects of landscape change on the spatial distribution of specialist and generalist herbivores across regions.

Rapid agricultural expansion and urbanization in China in the recent past have driven the conversion of the natural environment, such as forests and grasslands, into agricultural land and urban environments^20,21^. Shandong Province is a typical agricultural region in China. The maize (*Zea mays* L.) planting area increased by 49.35% from 2004 to 2016, reaching 3.67 million hectares^22^. Maize production in Shandong is constantly threatened by some insect pests. Here, we examine the effects of landscape simplification in Shandong Province on two herbivorous insects: the Asian Corn Borer [ACB, *Ostrinia furnacalis* (Guenee)] and Yellow Peach Moth [YPM, *Conogethes punctiferalis* (Guenee)]. Both species are lepidopteran pests in the grass moth family (Crambidae), and are key pests of maize causing damage by feeding on the ear. The ACB is one of the most significant pests of maize in Asia^23,24^. It causes 6 to 9 million tons of yield loss in an average year in China^25^. In most areas of Shandong, the ACB has three generations per year and overwinters as old larvae in maize stems. It can also feed on sorghum, millet, and cotton^23^. The YPM was originally considered to be a fruit pest, but it also attacks maize and is becoming increasingly prevalent in Chinese maize-growing regions^26^. In some areas of China, more YPMs than ACBs have been recorded in maize fields^27^. The YPM usually has three to four generations per year in North China, and overwinters as old larvae in maize stems^27^. There is niche overlap and interspecific competition between these pest species^28^. In field observations, it is common to see the coexistence of these two species on a maize plant. Although both the ACB and YPM utilize maize as a host plant, the species differ in their degree of resource specialization. We consider the ACB to be a specialist because it targets maize above other gramineous plants present in the study area. We treated the YPM as a generalist because it feeds on both maize and members of any other plant family found within the study area (see Supplementary Material Table S1 for its food sources in Shandong). We thus selected these two important pests to test for differences in specialist and generalist herbivores.

We used two levels of independent indicators to quantify the degree of landscape-wide agricultural simplification at each site. The first is the proportion of the area planted with maize in each county. The other is the proportion of cropland at the landscape scale surrounding each site, which has often been used as an indicator of landscape simplification or complexity^29,30^. The objectives of the current study were to (1) determine the spatial distribution patterns of the two pest species, the ACB and YPM, and (2) assess the relationship between pest abundance and landscape-wide agricultural expansion (proportion of maize in each county, proportion of cropland at the landscape scale). We predicted that the conditions associated with agricultural simplification could favor the specialist, the ACB, due to the increase in its resources. We also predicted that the generalist, the YPM, would benefit less from agricultural simplification.

## Materials and Methods

### Study sites

**Figure 1:**
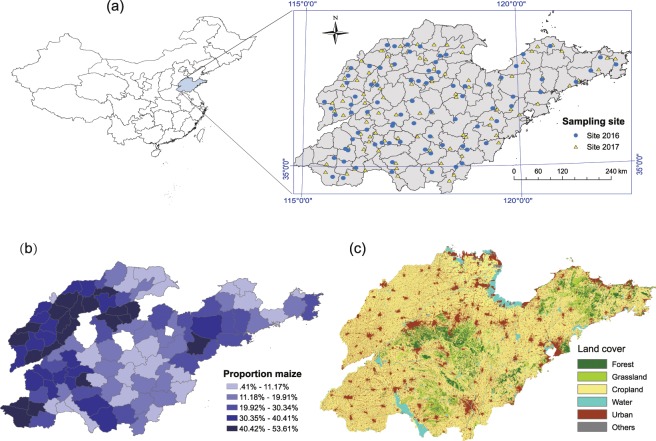
Map of the study area showing (**a**) the sampling location in Shandong Province, China; (**b**) the proportions of maize planting acreage (year 2017) in each sampling county; (**c**) land cover in the study area.

A two-year survey was conducted on maize in the landscape across 102 counties in Shandong Province, China (Fig. 1). The study region is located near the East Sea by the lower Yellow River (34°22.9′–38°24.0′N, 114°47.5′–122°42.3′E). This area has a continental monsoon climate featuring warm, humid summers and cold and dry winters. Precipitation is concentrated mainly in the summer months. The cropping system in this region mainly includes arable crops (wheat, maize, oil rape, and other crops) and greenhouses. The sampled 102 counties (average area ± SD: 1318 ± 516 km^2^) account for most of the total acreage of Shandong Province. We obtained information on the acreages of the maize planted in each county in 2016 and 2017 from the local agriculture bureau. The proportion of maize was calculated by dividing the maize acreage by the total acreage of each county. The proportion of planted maize ranged from 0.41% to 53.61% with a mean of 25.02% (Fig. 1b). The maize acreage was stable between these two years because the proportions of planted maize in these years were almost identical. Based on the precipitation data in recent decades (1981 to 2010) from the National Meteorological Information Center of China (http://data.cma.cn), the average monthly precipitation in this area is 172.8 mm in July and 152.0 mm in August. According to the above data source, in 2016, the precipitation values in July and August were both close to the records in previous years. However, in 2017, the precipitation was 17.8% and 7.1% higher in July and August, respectively, than in previous years.

### Insect sampling

Sampling was conducted three weeks prior to maize preharvesting. We randomly selected one maize field (area > 1000 m^2^) in each county based on a digital map. At each of the sampling fields, a “W”-pattern approach was used to sample insects. To avoid edge effects, sampling was conducted in the center of each field. We zigzagged down the field, stopping at 5 different locations. At each of these locations, 10 plants were cut open and examined. A total of 50 plants per field were sampled. Sampling began on 10 September 2016 (82 counties) and 20 September 2017 (97 counties). A total of 102 different counties were sampled. The insect abundances were measured on individual plants, which served as sampling units. The exact locations of the sampling sites were recorded using a Magellan® handheld GPS receiver (Magellan Navigation Inc., Santa Clara, CA), a latitude-longitude projection, and the WGS84 datum. Maize fields in the study area were maintained under conventional agronomic practices in terms of fertilization and herbicide application, and insecticides were applied as seed coating and spraying at the whorl stage of maize.

### Landscape composition analysis

Land-use maps of the sampling sites were obtained from a digital map provided by the Landsat 8 data with 30 m resolution collected in 2015. We used this imagery as our basic template to digitize the land use with ArcGIS 10 (ESRI, Redlands, CA, USA). We updated the original digital map by ground verification of the habitats present during the study period. Land-use types were classified into six general categories, including cropland, forest, grassland, water, urban areas, and other areas (Fig. 1c). Cropland was categorized as land primarily being cultivated with crops such as maize and cotton. Forest area included all forest and shrub land excluding the trees at residential sites. Grassland was herb-dominated land. Water represented wetlands and open water. Urban areas represented human architecture. Other areas included industrial area and roads, etc. We separately estimated landscape composition in three concentric circles of 1, 2, and 3 km radius from the center of each site. We used the proportion of cropland as an indicator of landscape simplification by crop expansion. In general, the cropland was 43.93% maize, 26.14% vegetables, 10.49% oil crops, and 19.44% other crops (see Supplementary Material Table S1).

### Data analysis

The spatial distribution patterns were assessed using Spatial Analysis by Distance Indices (SADIE) or SADIEShell (version 2.0)^31,32^. The software was developed for the spatial analysis of ecological data in the form of spatially referenced counts. SADIE determines the spatial characteristics of the observed arrangement of counts by comparing it to randomized permutations of the same counts among the sampling locations. SADIE finds the shortest total distance to regularity for the observed sample by moving the sampled ‘individuals’ between the sample points until the same number is achieved for each. After a number of randomizations, the distance to regularity can be calculated. In this way, a sample may be assigned an index of aggregation (*I*_*a*_) and a probability of aggregation (*P*_*a*_) based upon comparison of the observed distance to regularity with the distribution of permuted distances to regularity. Values of *I*_*a*_ in excess of unity denote spatial aggregation, those approximating unity indicate randomness and those less than unity indicate regularity^31^. The concepts underlying SADIE relate to a set of data as represented by regions, within which the observed counts are either arranged effectively at random or in clusters of units in two forms (patch/gap). A group of relatively high-density counts near one another is termed a patch cluster. A similar group of relatively small or zero counts is termed a gap cluster. In SADIE, the spatial pattern is measured locally, at each sampled unit, through an index of clustering. Each unit with a count greater than the overall mean is assigned a patch cluster index *V*_*i*_ (positive). Each unit with a count less than the overall mean is assigned a gap cluster index, which by convention is *V*_*j*_ (negative). Both indices have the associated probability. Once the degree of spatial pattern has been quantified locally, the patch (red) and gap (blue) cluster index for each sample unit may be contoured by interpolation to define patches (*V*_*i*_ > 1.5) and gaps (*V*_*j*_* < *−1.5), indicative of clustering half as large again as expected for random arrangements. Using this technique, red-blue plots were constructed for the ACB, the YPM and landscape variables (proportion of maize in county, proportion of cropland in landscape). Surfer 13 (Golden-Software Inc., CO, USA) was used to create distribution plots. The SADIE method has been further extended to assess the spatial association of two species^33^, on the basis of individual sampling unit clustering indices for each species. The measure of spatial association between the pest abundances and landscape variables was represented by the SADIE index, *χ*, the correlation coefficient between the clustering indices of the two sets. The *χ* index was calculated using the cluster indices of each dataset at a given site derived from the SADIE analysis. The measure *χ* of spatial association is based on a comparison of the spatial properties of each data set so that spatial features that match between the sets contribute to association and those that mismatch contribute to dissociation. A negative value generated from a sampling point (χ_p_) indicates a strong dissociation between the two datasets at that site, and positive values indicate a strong association. The mean comparison of χ_p_ for all sites provides an overall measure of the spatial association (χ) between the two datasets. If *p* < 0.025 there is a significant positive association, and if *p* > 0.975 there is a significant negative association.

Parametric techniques (linear models) were also used to analyze the influence of landscape variables on pest abundances. We performed linear regression using the “lm” function in R version 3.4.3^34^. According to previous studies on yield losses caused by the ACB^35^, we set the tolerable infestation level at 0.5 larvae per plant which corresponded to a 3–4% yield loss. Because of the lack of comprehensive studies on the YPM threshold, we assumed that the YPM causes a similar yield loss as the ACB, so the same tolerable threshold was also applied to the YPM. We evaluated the maize percentage values corresponding to these thresholds, if any linear relationships between pest number and maize percentage were found.

## Results

### Abundance

A total of 6215 larvae of the ACB (46.82%) and the YPM (53.18%) were found on 6900 maize plants during the two years. The ACB was more abundant in 2017 (0.47 ± 0.38 per plant) than in 2016 (0.30 ± 0.32 per plant) (F_1,177_ = 10.08, *p* = 0.002). In contrast, the YPM had similar abundances between the two years (2016: 0.50 ± 0.49 per plant; 2017: 0.46 ± 0.68 per plant, F_1,177_ = 0.146, *p* > 0.05) (all abundances are mean ± SD).

### Spatial aggregation

**Figure 2:**
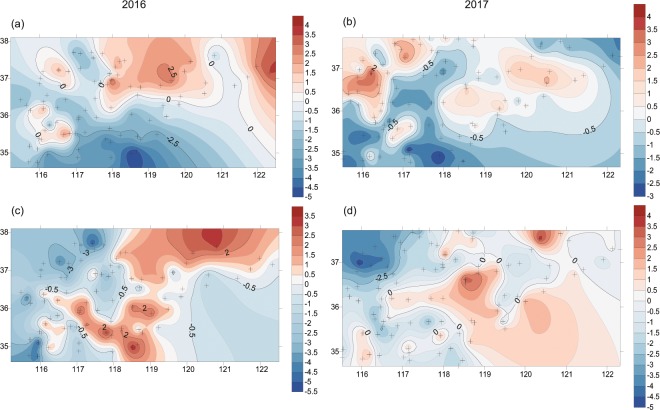
Spatial aggregation of the Asian corn borer (*Ostrinia furnacalis*) (**a,b**), and the yellow peach moth (*Conogethes punctiferalis*) (**c,d**). The contour maps represent interpolated (kriging) *V* cluster index of SADIE “red-blue” analysis for the pests and competition index in 2016 and 2017. Red filling refers to patch (*V*_*i*_ > 1.5), blue to gap (*V*_*j*_ < −1.5), (*p* < 0.05). The cross shows the geographic location of each site. The x-axis and y-axis represent longitude and latitude, respectively.

These two pests showed significant spatial aggregation in most cases (*I*_*a*_ > 1, *p* < 0.05), except for the aggregation of ACB in 2017 was marginally significant (Table 1, Fig. 2). The level of aggregation differed between the years. The aggregation of the ACB was stronger in 2016 (*Ia* = 2.041) than in 2017 (*Ia* = 1.385). The YPM was also aggregated more strongly in 2016 than in 2017 (Table 1). The spatial association between these pests differed between the years. In 2016, no association was found between the ACB and YPM (association index χ = −0.151, *p* < 0.975, not significant), but in 2017, they were negatively associated (i.e., segregated) (χ = −0.307, *p* = 0.998, significant).

**Table 1:** Spatial aggregation of the Asian corn borer (ACB) (*Ostrinia furnacalis*) and the yellow peach moth (YPM) (*Conogethes punctiferalis*) in our study area during 2016 and 2017.

Index	2016	*p*	2017	*p*
	Value		Value	
**ACB**				
*I*_*a*_	2.041	**0.002**	1.385	0.053
Patch (*V*_*i*_)	1.520	**0.028**	1.534	**0.024**
Gap (*V*_*j*_)	−1.824	**0.008**	−1.224	0.119
**YPM**				
*I*_*a*_	1.834	**0.003**	1.650	**0.014**
Patch (*V*_*i*_)	1.780	**0.007**	1.581	**0.020**
Gap (*V*_*j*_)	−2.010	**0.002**	−1.739	**0.012**

*I*_*a*_: average distance flow; *p*: associated probability, significant if *p* < 0.05 (in bold); *V*_*i*_*, V*_*j*_: cluster indices referring to patch and gap, respectively.

### Spatial associations between pests and the maize planting pattern

**Figure 3:**
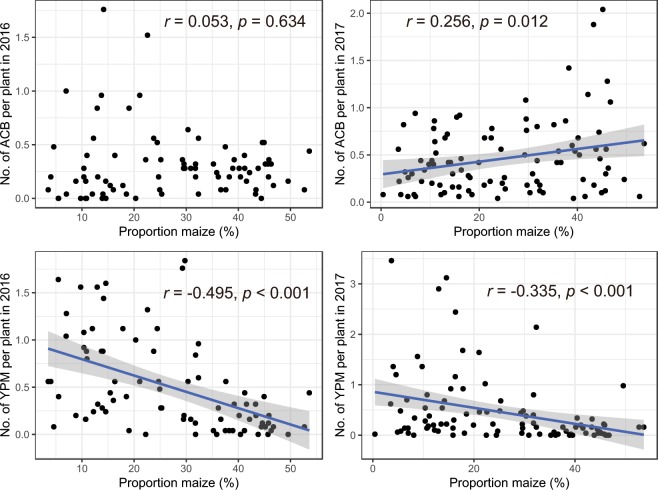
Abundances of the ACB and YPM in relation to the proportion of maize in each county. For significant relationships, regression lines are shown. (**a**) The ACB did not respond to the proportion of maize in county in 2016 (Pearson correlation *r* = −0.053, *p* = 0.634), while (**b**) the ACB increased with the proportion of maize in counties in 2017. Both (**c**) the YPM in 2016 and (**d**) the YPM in 2017 decreased with the proportion of maize in counties. The gray zone represents the 95% confidence interval of the abundance.

The spatial distributions of the two pests and the maize planting proportions in the different counties were significantly related, but the direction of the relationships differed between the species. The ACB was positively associated with the maize planting pattern (2016: χ = 0.188, *p* = 0.062, not significant; 2017: χ = 0.275, *p* = 0.004, significant); In contrast, the YPM was negatively associated with the maize planting pattern (2016: χ = −0.576, *p* > 0.999, significant; 2017: χ = −0.486, *p* > 0.999, significant). The effect of the proportion of maize in each county on ACB abundance was nonsignificant in 2016, but was significantly positive in 2017 (Table 2). ACB abundance was predicted to remain below the tolerable threshold when the proportion of maize was lower than 30.67% in 2017. YPM abundances were negatively correlated with the proportion of maize in both years (Table 2). YPM abundance was predicted to be below the threshold when the proportion of maize was higher than 27.15% (in 2016) or 22.69% (in 2017) (Fig. 3).

**Table 2:** Landscape effects on the Asian corn borer (*Ostrinia furnacalis*, ACB) and yellow peach moth (*Conogethes punctiferalis*, YPM): proportion maize in county, proportion of cropland at the 1–3 km radius.

Response	Predictors	df, n	F-statistic	*P*	r^2^
ACB in 2016	Maize	1, 80	0.23	0.634	0.003
	Cropland_1km	1, 80	0.06	0.807	0.001
	Cropland_2km	1, 80	0.05	0.824	0.001
	Cropland_3km	1, 80	<0.001	0.994	<0.001
ACB in 2017	Maize	1, 94	6.59	**0.012**	0.066
	Cropland_1km	1, 95	8.29	**0.005**	0.080
	Cropland_2km	1, 95	13.10	**<0.001**	0.121
	Cropland_3km	1, 95	11.39	**0.001**	0.107
YPM in 2016	Maize	1, 80	26.01	**<0.001**	0.245
	Cropland_1km	1, 80	0.12	0.735	0.001
	Cropland_2km	1, 80	0.41	0.525	0.005
	Cropland_3km	1, 80	0.06	0.809	0.001
YPM in 2017	Maize	1, 94	11.85	**<0.001**	0.112
	Cropland_1km	1, 95	5.90	**0.017**	0.058
	Cropland_2km	1, 95	10.32	**0.002**	0.098
	Cropland_3km	1, 95	12.36	**<0.001**	0.115

Displayed are degrees of freedom (df) of n replicates, F-statistic, *P*-values and r^2^ for each model. Significant *P*-values (*P* < 0.05) are highlighted in bold.

### Spatial associations between the pests and the proportion of cropland

Spatial associations between each of the pests and the proportion of cropland were significant mainly in 2017 (see Supplementary Material Table S2). The direction of the relationship was consistent at the landscape scale at a range of 1 to 3 km. In general, the ACB was positively related to cropland, whereas the YPM was negatively associated with cropland (Tables S2). The linear models also showed similar trends in 2017: ACB abundance was positively affected by the proportion of cropland, while YPM abundance was negatively affected by the proportion of cropland (Table 2).

## Discussion

The aim of this study was to determine the spatial patterns of the ACB and YPM and to identify the factors driving the spatial distribution of these two pests. We found that increased maize planting on the county level increased the likelihood of ACB aggregation and abundance but decreased YPM aggregation and abundance. Furthermore, the proportion of cropland (dominated by maize) in the landscape was also positively related to the distribution of the ACB but negatively related to that of the YPM, although these trends showed yearly variation. Devictor *et al*.^14^ showed that the spatial distribution of a given species in an anthropogenically degraded landscape may depend on the habitat specialization of the species. The spatial variation in the abundance of a given species is generally believed to reflect the extent to which local sites satisfy the species’ niche requirements^36^. There is evidence that specialist and generalist pests are not spatially distributed independently of each other. For instance, Julliard *et al*.^37^ showed the spatial segregation of specialists and generalists in bird communities. In our study, the ACB and YPM differed in their spatial distribution pattern, indicating that these two species have different ecological niches or patterns of habitat use.

Spatially aggregated abundance patterns can be driven by resource patterns^38^. Our hypothesis that agricultural simplification could favor the specialist ACB was confirmed. The spatial association analysis indicated that the aggregation of each species was related to the maize planting proportion. The pattern of maize planting matched the ACB aggregation pattern but did not match that of the YPM aggregation. Similarly, an increased proportion of maize in a county increased the ACB abundance and decreased the YPM abundance. For the ACB population, the presence of suitable host habitat should be the main determinant of its distribution. This is in accordance with the assumption that specialist herbivores benefit from larger host habitat areas. In contrast, landscapes simplified by maize planting were unfavorable for the YPM population. The YPM may depend on various habitat types in the landscape. The YPM has previously been described as a fruit pest. The typical reproduction habitats (e.g., orchards) for the YPM are likely to decrease in abundance with maize planting, which is indicated by negative correlation between woodland and cropland (Pearson’s r = −0.37, *P* < 0.001). The main reason why the YPM in maize fields was negatively affected by landscape simplification may be due to the loss of preferred habitat. On the other hand, the areas with many fruit trees could provide sufficient food for the YPM, which in turn may lead to the spillover of the YPM from orchards to maize fields.

A previous study showed that the ACB and YPM underwent interspecific competition and tended to use different parts of plants^28^. Our results showed that at a landscape scale, their spatial distributions were negatively associated. Both species were found to have significant relationships with maize plants. It is reasonable to infer that the spatial distributions of these pests are due to their difference in host use. Previous studies have addressed the life histories of these species individually^23,39^, but none of the studies in the literature have compared these two species under the same conditions. Based on field observations, we assume that the growth rate on maize might be similar between these two species. Both the maize proportion at the county level and the cropland proportion at the landscape scale showed consistent relationships with these pests. This finding suggests that the relationships between landscape simplification and pests were stable from a landscape scale to a larger scale (e.g., regional).

Many landscape ecology studies use the proportion of arable land (or semi-natural habitat) to indicate landscape simplification or complexity^40–42^. A meta-analysis showed that both specialist and generalist predators respond positively to landscape complexity^10^. However, the lack of differentiation between specialist and generalist pest responses to landscape complexity may be partly due to the chosen definitions of specialists and generalists. A specialist pest is often defined as being restricted to a particular family or genus of host plants, but may still find alternate hosts in a non-crop habitat^10^. We suggest that considering ecological specialization is important in the analysis of herbivores response to landscape change.

The yearly variation in ACB abundance may be partly due to weather, e.g. precipitation. The higher precipitation in 2017 indicates that 2017 was a wet year. However, the weather conditions in the sampling years still followed the trends in recent decades, as described in the Material and Methods section. Yearly variation in ACB populations is normal. Temperature variation at the sampling sites may also affect the population dynamics. Variation in the occurrence of pests can likely mask the relationships between pest and landscape factors and make them difficult to detect in some years. Therefore, long-term field scouting for pests is necessary. Host plants are usually an important factor affecting species abundance. Bt maize has not yet been commercially planted in China. Plant resistance to pests may be similar across locations. Other factors, e.g., pesticide use, can also affect pest abundance. In the study area, agriculture is dominated by smallholder farming^43^. The application of insecticides by farmers is usually focused on the whorl stage of maize. At the tassel and silk stages, farmers seldom use insecticides because spraying insecticides is cumbersome when traversing the fields and will increase labor costs. We assume that early insecticide input does not persist over the long term. The larvae that remained in the maize plants in preharvest period could reflect the actual damage level caused by these species. In view of controlling the ACB and YPM, more effective equipment and control strategies are needed at the tassel and silk stages. It is unknown whether these species have different performance in terms of pesticide resistance. Therefore, we cannot exclude the possibility that pesticide resistance or the biocontrol service provided by natural enemies may differentially affect the ACB and YPM. Our study focused on host plant factors and assessed their association with pest distribution. A shortcoming of this study is that more types of host habitats were not taking into account. Future studies should investigate the influence of the farming system (e.g., the composition of host plants) at a more refined scale.

As the ACB and YPM infestation levels were linearly related to the proportion of maize in a county or the landscape, we evaluated the threshold maize proportion that corresponded to tolerable economic thresholds of the pests. The maize-rich area (percent maize > approximately 30%) tended to experience more ACB pressure and less YPM pressure than areas with a lower percent maize. Because these two pests occur simultaneously at the reproductive stage of maize, control programs should consider both species. In landscapes with low maize proportions, farmers should keep close watch on the generalist YPM, while in maize-rich landscapes the specialist ACB is the biggest problem.

In conclusion, according to our results, agricultural expansion leads to higher infestation by a specialist pest, whereas the more generalist pest was negatively affected. This finding may help elucidate the spatial aggregation of two co-occurring pests on maize and their response to landscape simplification. Landscapes with large areas of maize tend to contain aggregated populations of ACBs but fragmented populations of YPMs. It appears that the distribution of the specialist depends on the host habitat areas, while that of the generalist depends on diverse habitats. These conclusions, based on a very large scale, should be tested in the future on a wider selection of specialist and generalist species and considering different landscape types.

## Supplementary information

Supplementary Information.
