# Supplementary material for: Landscape agricultural simplification correlates positively with the spatial distribution of a specialist yet negatively with a generalist pest
Source: Sci Rep. 2020 Jan 15;10:344. doi: 10.1038/s41598-019-57077-4 (PMC6962322; doi:10.1038/s41598-019-57077-4)
Supplement: Supplementary file 1 — Supplementary Information. [file 41598_2019_57077_MOESM1_ESM.docx]

**Landscape agricultural simplification correlates positively with the spatial distribution of a specialist yet negatively with a generalist pest**

Zhaoke Dong^1,2^ ^†^, Qingqing Zhang^1 †^, Lili Li^1^, Zengbin Lu^3^, Chao Li^1^, Fang Ouyang^4^, Teja Tscharntke^5^, Yi Yu^1^, Xingyuan Men^1*^

^1^ Institute of Plant Protection, Shandong Academy of Agricultural Sciences, Ji'nan 250100, China

^2^ Key Laboratory of Integrated Crop Pest Management of Shandong Province, College of Plant Health and Medicine, Qingdao Agricultural University, Qingdao 266109, China

^3^ Maize Research Institute, Shandong Academy of Agricultural Sciences, Ji'nan 250100, China

^4^ State Key Laboratory of Integrated Management of Pest Insects and Rodents, Institute of Zoology, Chinese Academy of Sciences, Beijing 100101, China

^5^ Agroecology, Department of Crop Science, Georg-August-University, Grisebachstrasse 6, 37077 Göttingen, Germany

^†^ These authors contribute equally to this work

^*^ Correspondence: Xingyuan Men, e-mail: menxy2000@hotmail.com.

**Table S1** The food resources of Asian corn borer (*Ostrinia furnacalis*) and Yellow peach moth (*Conogethes punctiferalis*) in Shandong province, China. The data of planting area was from Shandong Statistics Yearbook (2016).

| Category | Plants | Area (hectare) | Damaged by Asian corn borer | Damaged by yellow peach moth |
| --- | --- | --- | --- | --- |
| Food crops | Maize | 3173797 | √ | √ |
|  | Rice | 116280 |  |  |
|  | Millet | 16870 | √ | √ |
|  | Sorghum | 5213 | √ | √ |
| Economic crops | Vegetables | 1888555 |  | √ |
|  | Oil crops | 758260 |  | √ |
|  | Cotton | 515500 | √ | √ |
|  | Melons | 286759 |  |  |
|  | Tubers | 227000 |  |  |
|  | Beans | 151640 |  | √ |
|  | Herb | 33104 |  |  |
|  | Others crops | 27891 |  |  |
|  | Tobacco | 24359 |  |  |
| Fruits | Apple | 299675 |  | √ |
|  | Peach | 113163 |  | √ |
|  | Pearl | 45805 |  | √ |
|  | Grape | 43283 |  | √ |

**Table S2** Spatial association of pest abundances (*Ostrinia furnacalis*, ACB and *Conogethes punctiferalis*, YPM) to the proportion of cropland at 1–3 km scales (*χ*: association index; *p*: associated probability; significant if *p* < 0.025 or *p* > 0.975 (in bold).

|  | 1 km radius | 2 km radius | 3 km radius |
| --- | --- | --- | --- |
| ACB in 2016 |  |  |  |
| *χ* | 0.024 | 0.042 | 0.005 |
| *p* | 0.426 | 0.384 | 0.517 |
|  | Not significant | Not significant | Not significant |
| YPM in 2016 |  |  |  |
| *χ* | -0.096 | -0.331 | -0.257 |
| *p* | 0.805 | **0.999** | **0.991** |
|  | Not significant | Dissociation | Dissociation |
| ACB in 2017 |  |  |  |
| *χ* | 0.323 | 0.444 | 0.461 |
| *p* | **< 0.001** | **< 0.001** | **< 0.001** |
|  | Association | Association | Association |
| YPM in 2017 |  |  |  |
| *χ* | -0.206 | -0.360 | -0.241 |
| *p* | **0.975** | **> 0.999** | **0.988** |
|  | Dissociation | Dissociation | Dissociation |
